# New Adhesive Agent

**Published:** 1850-07

**Authors:** 


*New Adhesive Agent.*—M. Mellez, in an article published in the Bulletin de Thèrapeutique, of March 30th, recommends very strongly an adhesive preparation made of Shell lac dissolved in alcohol by the use of a moderate heat. The relative quantities of the articles should be sufficient to give the preparation the consistence of jelly or something approaching to it. It should be kept in a broad-mouthen bottle, corked to prevent desiceation. When wanted for use, it is to be spread upon strips of cloth of suitable size and form. M. Mellez enumerates its merits, contraction during its evaporation ; impermeability to air ; absence of all irritant action on the wound or skin ; intimate adherence to the skin ; resistance to the action of water, greasy matters, or the secretions from the wound ; and absence of any need of heat. In all these respects he asserts that it is superior to the collodion. It does not dry quite so quickly as the latter article, but it dries with sufficient rapidity to avoid any tax upon the patience of the surgeon. It does not require that the parts to which it is to be applied should be so thoroughly dried as does the collodion. It is also a much cheaper article. He asserts that from his observation he feels authorized to state that it is the most reliable and easy to manage of all the adhesive agents, that its agglutinative power resists the action of liquids and the moderate movements of patients, even when its application lasts for weeks ; that in drying it approximates the edges of the wound submitted to its action ; that the short time which elapses before the wound begins to cicatrize gives ground for a suspicion that it does something more than afford a mechanical protection ; and that in the treatment of fractures, especially such as are complicated with wounds, it is in every respect preferable to dextrine.—*Ibid.*

